# Trends of Microdiversity Reveal Depth-Dependent Evolutionary Strategies of Viruses in the Mediterranean

**DOI:** 10.1128/mSystems.00554-19

**Published:** 2019-11-05

**Authors:** Felipe Hernandes Coutinho, Riccardo Rosselli, Francisco Rodríguez-Valera

**Affiliations:** a Evolutionary Genomics Group, Departamento de Producción Vegetal y Microbiología, Universidad Miguel Hernández, Campus San Juan, Alicante, Spain; b Moscow Institute of Physics and Technology, Dolgoprudny, Russia; Dartmouth College

**Keywords:** metagenomics, microdiversity, marine, virome, depth gradient, virus, bacteriophage, evolution, Mediterranean, virology

## Abstract

The evolutionary interactions between viruses and their prokaryotic hosts remain a little-known aspect of microbial evolution. Most studies on this topic were carried out in pure cultures that challenge one virus with one bacterial clone at a time, which is very removed from real-life situations. Few studies have addressed trends of microdiversity in marine viral communities throughout depth gradients. We analyzed metagenomes from both the cellular and viral fractions of Mediterranean seawater samples spanning the epipelagic to the bathypelagic zones at depths of 15, 45, 60, and 2,000 m during the summer stratification of the water column. We evaluated microdiversity patterns by measuring the accumulation of synonymous and nonsynonymous mutations in viral genes. Our results demonstrated clear depth-dependent trends in the frequency of polymorphic sites and nonsynonymous mutations among genes encoding metabolic, structural, and replication proteins. These differences were linked to changes in energy availability, host and viral densities, and the proportions of actively replicating viruses. We propose the hypothesis that in the energy-rich, high-host-density, euphotic depths, selection acts to favor diversity of the host recognition machinery to increase host range, while in energy-depleted aphotic waters, selection acts on viral replication fitness, enhancing diversity in auxiliary metabolic genes.

**IMPORTANCE** Viruses are extremely abundant and diverse biological entities that contribute to the functioning of marine ecosystems. Despite their recognized importance, few studies have addressed trends of mutation accumulation in marine viral communities across depth gradients. By investigating these trends, we show that mutation frequencies differ among viral genes according to their molecular functions, with the highest microdiversity occurring among proteins related to host metabolism, followed by structural proteins and, lastly, genome replication proteins. This is in agreement with evolutionary theory that postulates that housekeeping genes are under strong purifying selection. We also observed a positive association between depth and microdiversity. One exception to this trend was the host recognition proteins from the deep chlorophyll maximum, which displayed strikingly high microdiversity, which we hypothesize to be associated with intraspecies competition for hosts. Finally, our data allowed us to propose a theoretical model for viral microdiversity across the depth gradient. These discoveries are of special relevance because many of the viral genomic sequences discovered here were predicted to infect some of the most abundant bacteria in marine ecosystems, such as “*Candidatus* Pelagibacter,” *Puniceispirillum*, and *Prochlorococcus*.

## Abstract (precis)

Viruses are extremely abundant and diverse biological entities that contribute to the functioning of marine ecosystems. Despite their recognized importance, few studies have addressed trends of mutation accumulation in marine viral communities across depth gradients. By investigating these trends, we show that mutation frequencies differ among viral genes according to their molecular functions, with the highest microdiversity occurring among proteins related to host metabolism, followed by structural proteins and, lastly, genome replication proteins. This is in agreement with evolutionary theory that postulates that housekeeping genes are under strong purifying selection. We also observed a positive association between depth and microdiversity. One exception to this trend was the host recognition proteins from the deep chlorophyll maximum, which displayed strikingly high microdiversity, which we hypothesize to be associated with intraspecies competition for hosts. Finally, our data allowed us to propose a theoretical model for viral microdiversity across the depth gradient. These discoveries are of special relevance because many of the viral genomic sequences discovered here were predicted to infect some of the most abundant bacteria in marine ecosystems, such as “*Candidatus* Pelagibacter,” *Puniceispirillum*, and *Prochlorococcus*.

## INTRODUCTION

Viruses are increasingly recognized as important players in the functioning of marine ecosystems (1, 2). In recent years, many efforts were undertaken to describe associations between viral biodiversity and spatial (3), temporal (4), and ecological (5) gradients. The taxonomic composition and functioning of host communities respond to such changes (6, 7). In response, viruses adapt to guarantee their survival. Previous studies have shown that depth and temperature are major factors structuring viral community composition (3, 8, 9). Viral macrodiversity is negatively correlated with depth, while microdiversity is positively correlated with it (8). In addition, viral communities from the bathypelagic zone display diverse auxiliary metabolic genes associated with nutrient uptake (5, 9) and have a preference for a lysogenic lifestyle (4). The depth gradient of stratified water masses displays marked changes in environmental conditions driven mainly by light availability and temperature (10). Thus, it is an ideal setup to study associations between environmental parameters, viruses, and their hosts.

In stratified waters, temperature decreases with depth, while the concentration of inorganic nutrients increases. The habitat at the thermocline provides photosynthetic microorganisms with ideal conditions of nutrient availability and light irradiation. The intense proliferation of photosynthetic microbes there leads to a peak of chlorophyll concentration and microbial cell density, known as the deep chlorophyll maximum (DCM). In the stratified water column, the DCM zone often exhibits the highest densities of prokaryotic cells and viral particles (11, 12). Moving toward the aphotic zone, the concentrations of inorganic nitrogen and phosphorus increase, but the gradual decrease of light hampers productivity; thus, both viral and bacterial abundances decrease, and the bathypelagic zone often displays the lowest densities of both bacteria and viruses (11, 13).

Previous studies have used metagenomics to assess changes in the taxonomic and functional compositions of viral communities throughout depth gradients (4, 14). Nevertheless, few studies have addressed patterns of microdiversity, i.e., accumulation of mutations within genomes, through the stratified water column (8, 15). Investigating patterns of microdiversity can help to elucidate the selective pressures acting upon viral genomes. For example, in coevolution experiments in which bacteriophages and hosts are cultured together over multiple generations, viruses tend to preferentially accumulate mutations in genes that affect their host range and the productivity of viral particles (16, 17). These discoveries provided insightful information regarding the processes by which viruses adapt to infect their hosts more efficiently in cultures, yet few studies have addressed this topic in free-living marine viral communities through culturing-independent approaches. These are necessary because the selective pressures acting on viral genomes in cultures and environmental communities might be drastically distinct.

Here, we sought to investigate microdiversity patterns in the environment to generate hypotheses about the selective pressures acting on marine viral communities throughout the depth gradient. We selected a site in the Mediterranean Sea off the coast of Spain during a period of water stratification (October 2015). Particularly in the Mediterranean, water temperature does not decrease below the seasonal thermocline, maintaining the same value of ca. 13°C all the way to the bottom, which makes the water column more homogeneous in terms of physical parameters (18).

**FIG 1:**
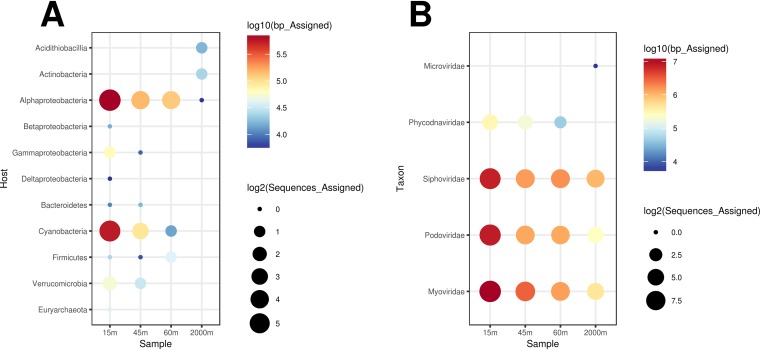
Taxonomic affiliations and predicted hosts of the bona fide viral scaffolds. (A) Bubble plot depicting host predictions obtained for viral scaffolds; (B) bubble plot depicting taxonomic assignments of the scaffolds based on percentages of matched proteins and the average amino acid identity to protein sequences from viral families in the NCBI nr database.

Seawater samples were retrieved from multiple depths, ranging from 15 (epipelagic zone), 45 (DCM zone), and 60 (DCM zone) m to 2,000 m (bathypelagic zone), and were used for preparing viral metagenomes (viromes). Viromes were assembled to obtain complete or partial viral genomes (Table 1 and Fig. 1). In parallel, we also analyzed cellular metagenomes from the same depth gradient. These had previously been assembled and binned to obtain metagenome-assembled genomes (MAGs) (10). Next, reads from both the viral and cellular fractions were mapped against the assembled viral scaffolds to calculate the level of microdiversity for each viral protein. Our rationale was that the changes taking place in microbial communities at the surface, in the DCM zone, and in aphotic habitats would subject the associated viral communities to different constraints, derived from the energy availability and host population density, which would be reflected in the microdiversity patterns within viral genomes.

**TABLE 1:** Characteristics of raw reads and assemblies of viral metagenomes

Depth (m)	No. of paired reads	Length (Gbp)	No. of scaffolds	*N*_50_ (kbp)	Maximum scaffold length (kbp)	Assembly size (Mbp)	No. of ORFs	Mean scaffold GC%
15	78,760,556	205.2	6,038	10.7	110.8	61.5	80,599	36.9
45	74,024,784	192.8	1,801	9	121.6	16.5	21,749	33.4
60	91,570,082	238.5	1,419	9.1	54.4	13	18,478	36.2
2,000	106,775,020	157.6	1,005	9.4	56.2	9.3	12,526	45.8

## RESULTS

### Environmental parameters and prokaryote community composition.

A previous study has analyzed cellular metagenomes and the metagenome-assembled genomes from this sampling site alongside the metadata on physical, chemical, and biological parameters necessary to characterize these water masses (10). We briefly summarize these results here to provide the framework in which the subject viral communities were found. Across the depth gradient (surface to 1,000 m), water temperatures ranged from 22.9°C to 13.1°C (Fig. S1; see also Table 1 in reference 10); surface waters had the highest temperatures, with intermediate temperatures in the DCM zone (45 m and 60 m) and the lowest temperatures in the bathypelagic zone (Table 2). Likewise, total organic carbon decreased with depth and ranged from 0.84 to 2.43 mg/liter. Nutrients such as total phosphorus (range, 0.1 to 0.45 μM), phosphate (0.06 to 0.39 μM), total nitrogen (0.4 to 8.89 μM), and nitrate plus nitrite (0.2 and 8.24 μM) followed the opposite trend and increased with depth; these variables displayed their highest values in the bathypelagic region. Ammonia concentrations displayed a different trend (range, 0.03 to 0.15 μM), with comparable values among the epipelagic zone samples (15 m, 45 m, and 60 m) and lower values for the bathypelagic samples. Chlorophyll *a* concentrations ranged from 0.01 to 0.78 mg/m^3^ and decreased with depth but peaked at 45 m (DCM zone). Cell abundances also varied with depth; specifically, the abundances of heterotrophic bacteria, *Synechococcus*, and *Prochlorococcus* peaked in the DCM zone (45 m) and continuously decreased toward deeper waters (10).

**TABLE 2:** Physical and chemical parameters throughout the depth gradient^*a*^

Depth (m)	Temp (°C)	Chlorophyll (mg/m^3^)	Oxygen (mg/liter)	TOC (mg of C/liter)	PO_4_^3–^ (μM)	Total P (μM)	Nitrate + nitrite (μM)	NH_4_^+^ (μM)	Total N (μM)	N/P ratio
15	22.9	0.1	7.09	2.43	0.06	0.1	0.2	0.13	0.4	4
45	15.8	0.78	9	1.46	0.1	0.14	0.25	0.14	0.48	3.43
60	14.5	0.36	7.66	1.43	0.08	0.12	0.23	0.15	0.46	3.83
1,000	13.1	0.01	6.1	0.84	0.39	0.45	8.24	0.03	8.89	19.76

a TOC, total organic carbon; total P, total phosphorus; total N, total nitrogen; N/P ratio, nitrogen-to-phosphorus ratio.

**FIG 2:**
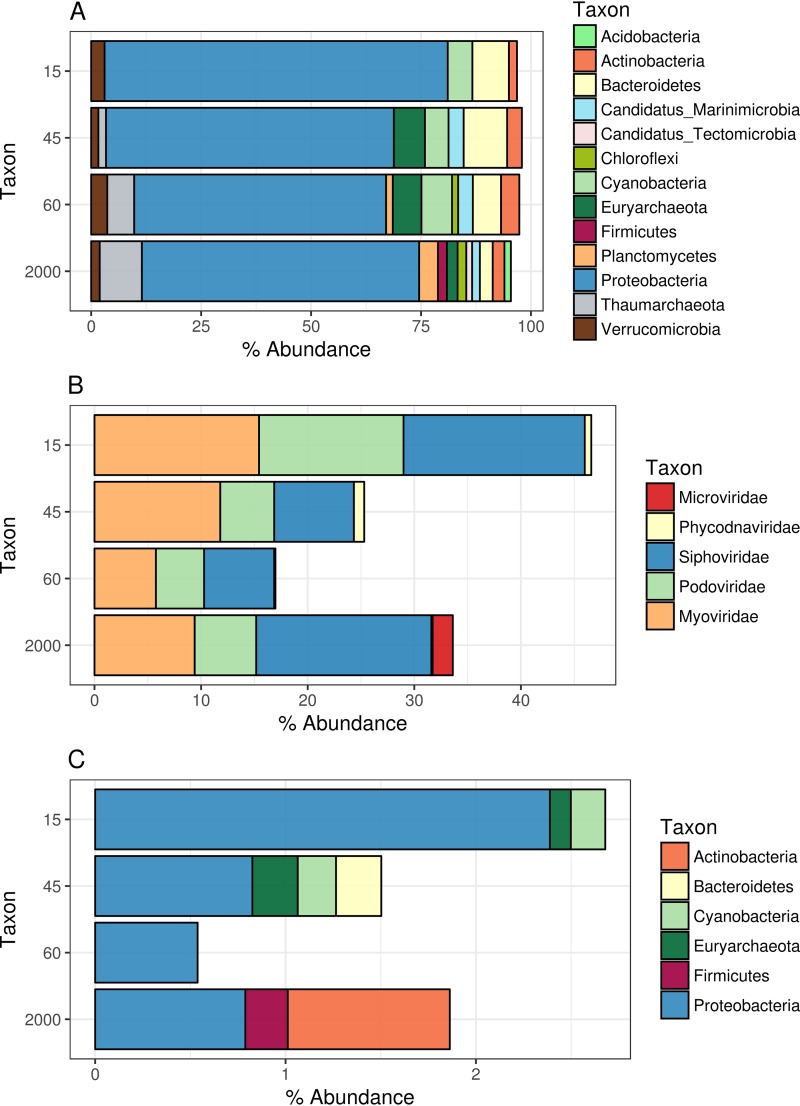
Prokaryote and viral community composition profiles across the depth gradient. Bar plots depict the relative abundances of prokaryote taxa or viruses. Only groups that displayed relative abundances equal to or above 0.1% are shown. (A) Prokaryote taxon abundances were calculated by querying reads from the cellular metagenomes against sequences in the NCBI nr database. (B) Viral abundances were calculated by querying reads against the database of assembled viral scaffolds and summing the percentages of reads mapped to the taxonomic classification of the scaffolds. (C) Same as for panel B but with viral abundances summed according to the putative host phylum of the scaffolds.

Similarly, the taxonomic compositions of the cellular fraction shifted across the depth gradient (Fig. 2A). At all depths, communities were dominated by the *Proteobacteria* (mostly from the class *Alphaproteobacteria*, followed by *Gammaproteobacteria*), but the abundances of these groups shifted throughout the depth gradient. The abundances of *Alphaproteobacteria* and *Cyanobacteria* decreased with depth, while the abundances of *Euryarchaeota* and *Thaumarchaeota* followed the opposite trend. Other groups that were detected in these samples at comparable abundances throughout the depth gradient were *Beta*- and Deltaproteobacteria, *Marinimicrobia*, *Actinobacteria*, *Bacteroidetes*, *Chloroflexi*, *Planctomycetes*, and *Verrucomicrobia*.

### Assembled viral genomes and predicted hosts.

Assembly of viral metagenomes yielded 10,263 genomic sequences of lengths equal to or greater than 5 kbp, within which 133,352 open reading frames (ORFs) were identified (Table 1). A total of 7,164 (69.8%) scaffolds were classified as bona fide viral sequences (see Materials and Methods). Among these, 21 scaffolds with lengths equal to or above 10 kbp (average length, 44 kbp) and with overlapping ends, which likely represent complete viral genomes, were identified. The percentages of virome reads that mapped to these bona fide viral scaffolds were 75.1% for the 15-m sample, 45.7% for the 45-m samples, 55.38% for the 60-m samples, and 59.7% for the 2,000-m sample.

Computational host predictions were obtained for the bona fide viral sequences by scanning viral and prokaryote genomes for three signals of virus-host association: homology matches (i.e., long genomic segments sharing high nucleotide identity), shared tRNA genes, and matches between CRISPR spacers and viral sequences. These approaches have previously been benchmarked and shown to provide accurate host predictions, especially at higher taxonomic ranks, such as phylum and class (19, 20). In addition, we manually curated host predictions by investigating the gene contents of viral genomic sequences. Host predictions were obtained for 171 of the bona fide viral sequences (see Table S1 in the supplemental material and Fig. 1A). Among those, the majority were predicted to infect *Proteobacteria* (99 sequences), particularly *Alphaproteobacteria* of the genera “*Candidatus* Pelagibacter” (52 sequences) and *Puniceispirillum* (38), followed by *Cyanobacteria* (58) of the genera *Prochlorococcus* and *Synechococcus*.

10.1128/mSystems.00554-19.3 TABLE S1 Detailed description of the assembled virome sequences, including sequence ID, length, number of ORFs, sample source, predicted host, recruitment criteria, taxonomic classification, VirSorter category, and VirFinder score. Download Table S1, XLS file, 3.9 MB. Copyright © 2019 Coutinho et al. 2019 Coutinho et al. This content is distributed under the terms of the Creative Commons Attribution 4.0 International license.

Taxonomic classification of the assembled scaffolds identified most of them as tailed bacteriophages from the families *Myoviridae*, *Podoviridae*, and *Siphoviridae* (Fig. 1B). In addition, some of the scaffolds from the epipelagic samples were classified as *Phycodnaviridae*, viruses that infect eukaryotic algae. Scaffolds annotated as *Microviridae* bacteriophages were exclusively retrieved form the bathypelagic sample.

### Viral community composition.

The taxonomic compositions of viral communities also displayed shifts according to depth (Fig. 2B). The families of tailed bacteriophages of the families *Myoviridae*, *Podoviridae*, and *Siphoviridae* were dominant in all samples and together accounted for 15% to 45% of the annotated reads. Eukaryotic viruses from the family *Phycodnaviridae* were detectable only in the epipelagic samples, although at lower abundances. Bacteriophages from the family *Microviridae* were abundant in the bathypelagic sample only. The higher abundance of this family of single-stranded DNA (ssDNA) viruses in this sample is likely an indication that viruses with less complex and smaller genomes are favored under the conditions of low energy availability that are typical of the bathypelagic zone.

Grouping viral scaffold abundances according to predicted hosts revealed differences among samples of the depth gradient (Fig. 2C). Scaffolds predicted to infect *Proteobacteria* were among the most abundant in all depths, with abundances ranging from 0.5% to 2.4% of mapped reads. Scaffolds predicted to infect *Cyanobacteria* and *Euryarchaeota* displayed their highest abundances in the 15-m and 45-m samples, while those predicted to infect *Bacteroidetes* were abundant only in the 45-m sample. The 2,000-m virome displayed a unique profile, with scaffolds predicted to infect *Firmicutes* and *Actinobacteria*, albeit at abundances below 1%. Although hosts could be assigned only to a small fraction of the scaffolds, their host-grouped abundance patterns were in agreement with what was expected, considering the observed prokaryote community composition (Fig. 2A).

### Mediterranean viruses actively replicating in the cellular fraction.

**FIG 3:**
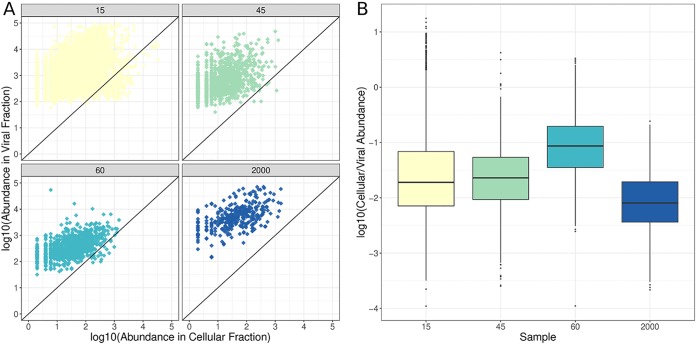
Viral scaffold abundances in viral and cellular metagenomes from the depth gradient. (A) Scatterplots depicting the relative abundances of viral scaffolds in the viral (*y* axis) and cellular (*x* axis) metagenomes. Each dot represents one of the viral scaffolds assembled from a respective virome. (B) Boxplots depicting the ratio of the abundances of viruses in the cellular and viral fractions for each sample. Boxes depict the median and the first and third quartiles. Whiskers extend to 1.5 times the size of the interquartile ranges. Outliers are represented as dots above or below whiskers.

Read mapping revealed that many of the viral scaffolds assembled from viromes could also be detected in the cellular metagenomes, some in very high abundances (Fig. 3A). These cellular metagenomes were prepared aiming to extract DNA from cells only, while free viral particles were expected not to be retained by the 0.22-μm filter, unless they were inside prokaryotic cells. Also, viral genomes are much smaller than those of prokaryotes; hence, their abundance in cellular metagenomes is expected to be very low unless many copies of their genomes are present. Based on that, we assumed that the viral sequences that were abundant in the cellular metagenomes are derived from actively replicating viruses undertaking lytic infections, which leads to high copy numbers of their genomes inside host cells and to their high abundances among the cellular metagenomes (20, 21). Alternatively, viral sequences in the cellular fraction may be the result of lysogenic infections, yet those are not expected to produce the high copy numbers of viral genomes inside host cells that would be necessary to yield high abundances of viral genomes in the cellular metagenomes.

Abundances of the assembled viral scaffolds in the cellular fraction differed between samples. The average ratios of cellular- to viral-fraction scaffold abundances were highest for the 45- and 60-m samples, followed by the 15-m and lastly the 2,000-m sample (Fig. 3B). We also analyzed the taxonomic affiliations of raw reads from cellular metagenomes by querying them against the NCBI nonredundant (nr) database. This result also pointed to the highest abundance of viruses in the cellular-fraction samples from the DCM zone (45 m and 60 m). These data suggest that there were more viruses actively replicating at the DCM zone, followed by the 15-m sample and lastly the 2,000-m sample, which displayed the lowest proportion of actively replicating viruses. In addition, the DCM virome samples displayed the lowest values for the Shannon diversity index (5.55 and 5.61 at 45 and 60 m, respectively), while these values were higher for the 15-m (7.21) and 2,000-m (7.26) samples. The high proportion of actively replicating viruses and the low Shannon diversity (calculated based on the abundances of viral scaffolds) observed in the DCM zone suggest that the intense viral replication taking place at these depths leads to a highly clonal community, with many nearly identical viral genomes coexisting at high densities.

### Levels of microdiversity shift throughout the depth gradient and across functional categories.

We evaluated microdiversity patterns by measuring the percentage of polymorphic sites and the ratios of the number of nonsynonymous polymorphisms per nonsynonymous site to the number synonymous polymorphisms per synonymous site (*pN/pS* ratios) of protein-encoding genes identified in the bona fide viral scaffolds. The *pN/pS* ratio is a measure analogous to the *dN/dS* ratio (a ratio of the number of nonsynonymous to synonymous mutations); it does not require specific haplotypes to be identified and, therefore, can be applied to metagenomic data sets to provide a population-level measure of microdiversity (22–24). Briefly, reads from the metagenomes were mapped to the assembled scaffolds using the sensitive local mode of Bowtie2 (25). Next, mapped reads were used to detect mutations (i.e., differences between reads and assembled scaffolds), specifically, single nucleotide polymorphisms (SNPs). Next, *pN* and *pS* were calculated by, respectively, dividing the observed counts of nonsynonymous and synonymous mutations by the expected frequencies of these mutations under a neutral model (see Materials and Methods for further details).

**FIG 4:**
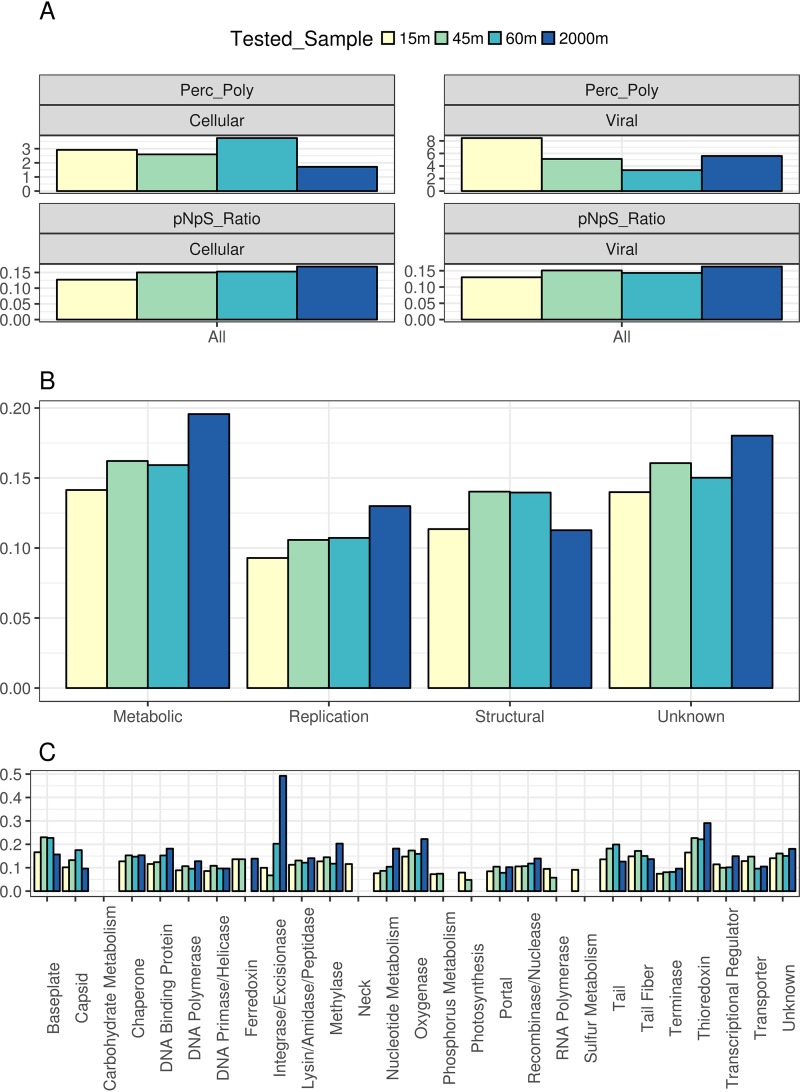
Polymorphic sites and *pN/pS* values of viral genes differ among depths and functional categories. (A) Bar plots depict the median percentage of polymorphic sites (Perc_Poly) and the median *pN/pS* values of all the ORFs derived from viral scaffolds in the cellular and viral fractions, regardless of gene function. (B and C) Median *pN/pS* values of ORFs derived from viral scaffolds grouped by sampling site and broad functional category (B) and grouped by sampling site and specific functional category (C) for the viral fraction only. Only proteins derived from the set of bona fide viral sequences were included in these analyses. When we calculated medians, only proteins that displayed *pN* and *pS* values above 0 were included. Also, only proteins with a total number of polymorphic sites equal to or above 1 and a percentage of polymorphic sites equal to or above 1% were included so as to avoid estimating *pN/pS* values based on only a small fraction of protein lengths. Median values obtained from fewer than three proteins were omitted.

The median percentages of polymorphic sites in viral proteins ranged between 1% and 8%; these medians were notably higher in the viral-fraction metagenomes (Fig. 4A). Among the viral metagenomes, that at 15 m displayed the highest median percentage of polymorphic sites, followed by those of the 2,000-m, 45-m, and, lastly, the 60-m sample. The higher frequency of polymorphic sites observed in the 15-m sample might be a consequence of the higher intensity of UV radiation near the surface. Meanwhile, the lowest percentage of polymorphic sites observed in viromes from the DCM zone (45-m and 60-m samples) corroborates our finding that these are highly clonal viral communities. A different pattern was observed among the cellular metagenomes: the 60-m samples displayed the highest median percentage of polymorphic sites, followed by the 15-m, 45-m, and, lastly, the 2,000-m samples. The overall low percentage of polymorphic sites means that the observed microdiversity was concentrated in few amino acids within protein-encoding genes. Thus, the observed mutations were restricted to microdiversity hot spots within viral proteins.

The majority of proteins displayed *pN/pS* values below 1, regardless of the sample or fraction, meaning that the frequencies of nonsynonymous mutations was below that which was expected by chance. Thus, purifying selection was a major driving force regulating frequencies of mutations among viral genes. Nevertheless, 117 proteins (0.088% of the total) displayed *pN/pS* ratios above 1 in the cellular-fraction metagenomes and 1,092 proteins (0.819%) in the viral-fraction metagenomes. Most of these proteins were retrieved from the 15-m sample (755 proteins, 0.94% of the proteins obtained at this depth), followed by the 2,000-m (239, 1.1%), 45-m (148, 0.8%), and 60-m (67, 0.53%) samples. Although the majority of them had no assigned functions, some of these proteins were identified as recombinase/nucleases (*n* = 21), oxygenases (*n* = 17), lysins (*n* = 16), methylases (n = 13), and tail fibers (*n* = 11).

We observed a positive association between depth and the median *pN/pS* ratio of each sample (Fig. 4A). The highest median of *pN/pS* value was observed for the 2,000-m sample, followed by the 45-m, 60-m, and, lastly, the 15-m sample. This association between *pN/pS* ratio and depth was observed in the metagenomes from both the viral and cellular fractions. Because the coverage of proteins in the viral-fraction metagenomes was much higher and spanned many more of the viral proteins with a higher percentage of polymorphic sites, we focused subsequent analysis of *pN/pS* ratios on the viral-fraction metagenomes only.

Due to the many unknown proteins present in marine viral genomes, our capacity to annotate these genes and predict their function is limited (26). Nevertheless, we observed marked differences of median *pN/pS* ratios among proteins according to functional categories (Fig. 4B and C). Genes involved in genome replication (e.g., DNA polymerase, DNA primase, and nucleotide metabolism genes) displayed the lowest median *pN/pS* values compared to those of other categories. Structural viral proteins (e.g., capsid, neck, and tail) showed intermediate median *pN/pS* values. Finally, proteins associated with altering host metabolism (e.g., ferrochelatases, thioredoxins, and oxygenases) displayed the highest median *pN/pS* values. The positive association between the *pN/pS* ratio and depth was also observed when we grouped proteins according to broad functional categories (Fig. 4B). A notable exception was the median *pN/pS* ratio of structural proteins, which was highest for the DCM samples.

These differences of *pN/pS* ratios among functional categories are associated with the roles of genes during the viral infection cycle. Genes involved in genome replication must operate at high fidelity and efficiency; thus, deleterious nonsynonymous mutations in these proteins are readily removed from the population by purifying selection. Meanwhile, structural proteins are fundamental for adequate particle assembly, encapsulation of the viral genome, and host recognition. Deleterious mutations in structural genes can also compromise viral infections, but not as much as errors during genome replication. Finally, metabolic genes are responsible for redirecting host metabolism toward pathways that favor viral-particle production (27, 28). Thus, lower efficiencies of metabolic genes due to deleterious mutations are likely to reduce viral productivity but not to compromise it as much as deleterious mutations in genome replication or structural modules.

The associations between microdiversity and depth, as well as the differences among the functional categories described above, were also observed when we used stricter read mapping criteria (very fast mode in Bowtie2 and subsampling to 10 million reads). Therefore, it is unlikely that our observations regarding the differences among depths and gene functions are the result of spurious read mapping due to permissive cutoffs. Also, we detected only weak associations between the percentage of polymorphic sites or *pN/pS* ratios with the coverage of the assembled scaffolds (Pearson correlation scores = 0.14 and 0.05, respectively), meaning that scaffolds with higher coverage did not necessarily have more SNPs reported. This demonstrated that the observed differences in the percentages of polymorphic sites or *pN/pS* ratios among samples were not merely a consequence of the different numbers of reads mapped to each scaffold.

### Differences among depths and functional categories are also observed in independent data sets.

We sought to determine whether the differences observed among these Mediterranean Sea samples regarding the microdiversity of different depths and functional categories also occurred in other regions of the global ocean. Therefore, we analyzed samples from two data sets for which sampling was performed throughout depth gradients and included samples from the surface, the DCM zone, and the bathypelagic zone. Specifically, we analyzed virome samples from the Tara Oceans expeditions (8) collected in the Indian Ocean and samples from the ALOHA (a long-term oligotrophic habitat assessment) (4) expeditions in the Pacific Ocean. For the latter, only metagenomes from the cellular fraction were available.

Overall, we observed that the results obtained from these three independent data sets were in agreement (Fig. S2). First, the positive association between depth and *pN/pS* ratios was also observed for the ALOHA and Tara Oceans data sets. Second, the differences in functional categories which pointed to the highest *pN/pS* ratios for metabolic proteins, followed by structural and, lastly, genome replication proteins, were also observed in the *Tara* data set (the ALOHA samples did not have sufficient coverage to reliably calculate *pN/pS* medians per functional categories). Third, the higher *pN/pS* ratios in the DCM zone observed for structural proteins (specifically, tail proteins) were observed in the Tara Oceans data set. Together, these results indicate that the differences in *pN/pS* ratios seen among depths and functional categories for the Mediterranean samples are not exclusive to this site. Instead, similar patterns were observed in independent data sets from the Pacific and Indian Oceans.

10.1128/mSystems.00554-19.1 FIG S1 Conductivity-temperature-depth (CTD) data depicting the associations between depth, temperature, and chlorophyll *a* concentrations for the depth profile analyzed in this study. Download FIG S1, PDF file, 0.08 MB. Copyright © 2019 Coutinho et al. 2019 Coutinho et al. This content is distributed under the terms of the Creative Commons Attribution 4.0 International license.

10.1128/mSystems.00554-19.2 FIG S2 Percentage of polymorphic sites and *pN/pS* values of viral genes differ among depths and functional categories for the Tara Oceans (DCM at 65 m) and ALOHA (DCM at 125 m) data sets. Data was obtained using metagenomes from the cellular fraction for the ALOHA metagenomes and in the viral fraction for Tara Oceans viromes. (A) Bar plots depict the median percentage of polymorphic sites and the median *pN/pS* values of all the ORFs derived from viral scaffolds, regardless of function in the Tara Oceans data set. (B) Median *pN/pS* values of ORFs derived from viral scaffolds grouped by depth and broad functional category in the Tara Oceans data set. (C) Median *pN/pS* values of ORFs derived from viral scaffolds grouped by depth and specific functional category in the Tara Oceans data set. (D) Bar plots depict the median percentage of polymorphic sites and the median *pN/pS* values of all the ORFs derived from viral scaffolds, regardless of function in the ALOHA data set. When calculating medians, only proteins that displayed *pN* and *pS* values above 0 were included. Also, only proteins with a total number of polymorphic sites equal or above 1 and percentage of polymorphic sites equal or above 1% were included, so to avoid estimating *pN/pS* values based only on a small fraction of protein length. Median values obtained from less than three proteins were omitted. Download FIG S2, PDF file, 0.5 MB. Copyright © 2019 Coutinho et al. 2019 Coutinho et al. This content is distributed under the terms of the Creative Commons Attribution 4.0 International license.

### The DCM is a microdiversity hot spot for viral receptor binding proteins.

The DCM samples displayed the highest median *pN/pS* values for structural proteins (Fig. 4B). Specifically, structural proteins of baseplate, capsid, tail, and tail fiber genes displayed median *pN/pS* values higher than those of their counterparts in the remaining samples (Fig. 4C). Interestingly, all of these proteins either are or interact directly with receptor binding proteins that mediate host recognition, a fundamental step for successful viral infection (29, 30). The enhanced *pN/pS* ratios observed for these genes in the DCM zone provides evidence that this habitat is a microdiversity hot spot for viral receptor binding proteins.

**FIG 5:**
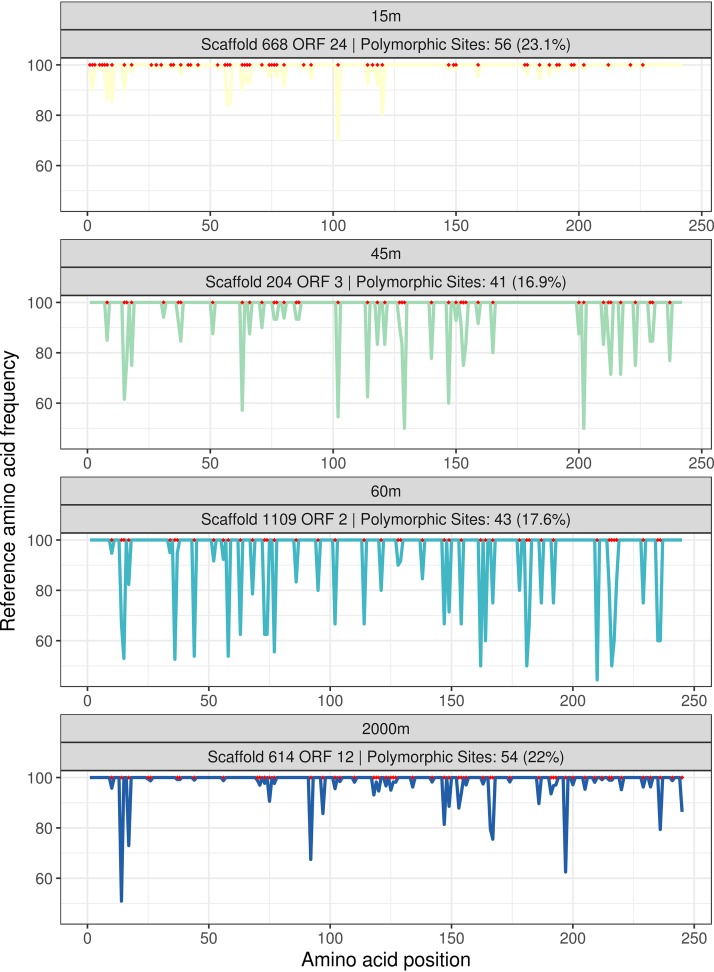
Occurrence of nonsynonymous mutations among a group of homologous tail proteins. These proteins are homologous and annotated as tail proteins (which are involved in host recognition). Each of them (ORF 24 from scaffold 668 in the 15-m virome, ORF 3 from scaffold 204 in the 45-m virome, ORF 2 from scaffold 1109 in the 60-m virome, and ORF 12 from scaffold 614 in the 2,000-m virome) was retrieved from scaffolds assembled at different depths, thus allowing for the comparison of the patterns of accumulation of nonsynonymous mutations at each analyzed depth among homologous proteins. In the line plots of the bottom panel, the *x* axis depicts the amino acid position along proteins, and the *y* axis depicts the frequency of the reference amino acid (i.e., the most frequent) among the reads from each sample. The red dots along the *x* axis indicate which sites throughout the protein are polymorphic sites. Valleys in the plot represent areas that concentrate nonsynonymous mutations, possibly driven by positive selection favoring mutations that modify or expand host range.

Adaptation to suboptimal hosts is a major driver of genomic diversification for viruses, which is associated with the quick accumulation of nonsynonymous mutations in tail fiber proteins (17). A single nucleotide polymorphism in the tail fiber gene can be sufficient to alter viral host range (16, 31). Consistently with those findings, we observed multiple cases of tail proteins in which nonsynonymous mutations were concentrated in small segments of these genes (Fig. 5). The sites that accumulate nonsynonymous mutations at higher frequencies than the other codons are likely those that confer a selective advantage to the virus in its specific habitat according to the availability of hosts. These trends are consistent with a scenario in which, on the one hand, positive selection acts on tail fiber proteins to expand host range and, on the other hand, purifying selection removes mutations from sites where they cause loss of function or restrict the host range instead of expanding it (17, 32).

## DISCUSSION

### Different selective pressures determine levels of microdiversity throughout the depth gradient.

For the kind of analysis that we have performed, it is critical that the mapped reads used as input for *pN/pS* ratio calculation come from the same viral population. Actually, we sought to cluster the assembled scaffolds from each sample into viral populations as defined in references 3, 8, and 33. This was performed individually for each sample, and we found that only 8 out of the 7,164 (0.11%) scaffolds could be clustered into viral populations. Therefore, we assume that each assembled scaffold represents the consensus sequence of a single viral population residing at each of the sampled depths. Thus, we interpret our results based on the rationale that the per-scaffold analysis is representative of the per-population trends of microdiversity and so are the obtained values for the percentage of polymorphic sites and the *pN/pS* ratio.

**FIG 6:**
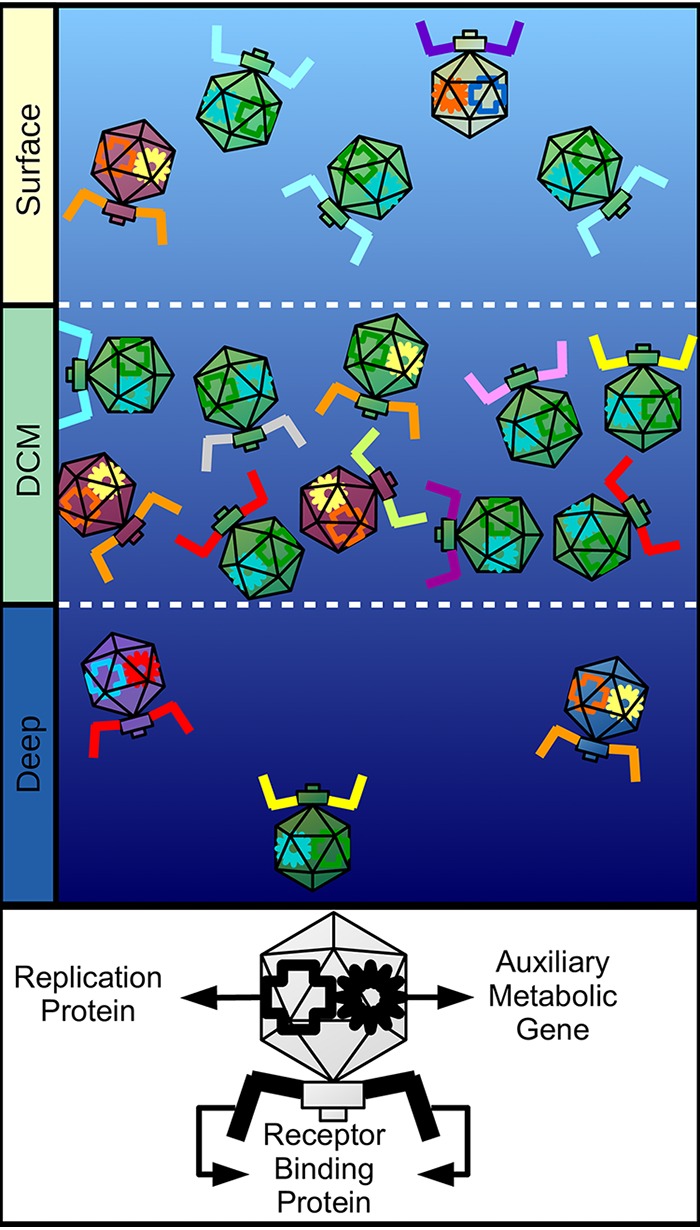
Conceptual model summarizing the observed patterns of microdiversity in marine viral genomes across the depth gradient. Different capsid colors represent different viral species. Different colors for receptor binding proteins, auxiliary metabolic genes, and replication proteins represent different isoforms of the same protein created by nonsynonymous mutations. Surface samples have intermediate densities of viral particles and intermediate species diversity; these samples displayed the lowest degree of microdiversity for all functional categories. DCM samples have the highest density of viral particles but the lowest species diversity; these samples displayed the highest degree of microdiversity among receptor binding proteins. Deep samples have the lowest density of viral particles but the highest species diversity; these samples displayed the highest degree of microdiversity among metabolic and replication proteins.

Major changes take place among prokaryotic and viral communities throughout the depth gradient, affecting their taxonomic composition and cell densities (4, 10, 11, 34). These differences impact viral microdiversity because the rate at which viral genomes accumulate mutations is replication rate dependent, so that they adapt faster under conditions with a higher host density, in which more infection events take place (35). Our results demonstrated that the DCM viral communities had the highest proportions of actively replicating viruses but were the least diverse (according to their Shannon index). We propose that this scenario leads to intense intraspecies competition between viruses for suitable hosts, creating a selective pressure that favors viruses with mutations in receptor binding proteins which provide them with a different host range, allowing them to exploit a distinct niche (Fig. 6). A recent global-scale study of marine viral communities found that their degree of microdiversity is significantly associated with depth and temperature (8). Our results agree with those findings but also expand them by demonstrating differences in specific functional categories across each depth and linking them to environmental conditions and host communities.

The high microdiversity observed among receptor binding proteins and the clonal populations observed within DCM samples suggest that many strains of viruses with distinct host ranges coexist in this habitat. It follows that host strains with different patterns of viral susceptibility also coexist in these sites. This is in agreement with the constant-diversity model (36), which proposes that complex multiclonal populations of viruses and cells are required for optimal ecosystem functioning (16, 32).

Meanwhile, a different pattern was observed for the bathypelagic sample. In this habitat, both viral and cell densities are much lower than in the DCM zone or near the surface (11, 13). Therefore, chance encounters between viruses and hosts are expected to occur less often. Thus, fewer infection events take place at 2,000 m than in shallower depths, with the higher host densities, as evidenced by the differences in abundance of viruses actively replicating in the cellular fraction. Interestingly, the bathypelagic sample displayed the lowest proportion of actively replicating viruses but the highest Shannon diversity index. This finding suggests that in the energy-limited bathypelagic zone, intraspecies competition for hosts is expected to be less relevant than it is in the DCM zone, where a highly clonal population with high density was observed. Instead, the major constraint faced by viruses at this depth might be the efficient production of viral progeny, since in this scenario, a lower reproductive fitness is more likely to lead to local extinction. Consistently with that, we observed the highest *pN/pS* values of proteins encoding metabolic functions (e.g., oxygenases and thioredoxin) and transcriptional regulators in the 2,000-m sample. We postulate that the higher microdiversity observed among these genes in the bathypelagic sample is evidence of positive selection acting on proteins that increase the capacity of viruses to generate progeny; the viruses use a diverse array of auxiliary metabolic genes and transcriptional regulators to fine-tune host metabolism to enhance the production of viral particles under conditions of low energy availability and productivity (Fig. 6). These special characteristics of viruses in the bathypelagic sample might be linked to the higher diversity of auxiliary metabolic genes seen among viral communities from these depths (5, 37) and to the prevalence of a lysogenic lifestyle among them (38, 39). Together, these differences in viral lifestyle might contribute to the higher observed microdiversity of metabolic genes.

### Microdiversity patterns differ between pure cultures and environmental samples.

In laboratory experiments of phage-bacterium coevolution, mutations usually accumulate in genes involved in host specificity, such as tail fiber proteins (17, 40). In contrast, we observed a broad distribution of mutations that spanned all functional categories within viral genomes. We attribute this to the differences between the selective pressures imposed on viruses in coevolution experiments versus in the natural environment. In the former, the only selective pressure is to effectively infect a single host derived from a clonal population. In the latter, viruses have a multitude of hosts available, each with their specific viral receptors and resistance mechanisms (e.g., CRISPR and restriction modification systems). In cultures, once resistance mutations appear, their prevalence quickly rises within bacterial populations (17). In the environment, the frequency of resistance mutations is simultaneously regulated by a trade-off viral resistance and the fitness cost brought by these resistance mutations (32). These differences in selective pressures faced by viruses in environmental communities likely lead to the accumulation of mutations throughout the entirety of viral genomes, not just at the sites associated with host recognition and infection. Furthermore, in laboratory experiments, the microdiversity observed is derived from a single viral population, whereas our results, retrieved from environmental data, likely represent a consensus of all the viral populations coexisting at each of the sampled depths. The wealth of information provided by metagenomic data sets may shed light on the real paths of coevolution that take place between the diverse populations of bacteria and viruses that are found in nature.

### Caveats and limitations.

Some matters must be taken into consideration when interpreting our results. First, we used multiple-displacement amplification (MDA) to generate viral metagenomes. This was necessary given the low yields of the viral DNA extraction step, which made the samples inadequate for sequencing as they were. The use of MDA is a common practice in the preparation of viral metagenomes (41–43). Nevertheless, MDA is known to introduce some bias in virome preparation. Specifically, not all DNA is amplified equally, making the obtained viromes not as quantitative as those obtained without this approach. Due to this uneven amplification phenomenon, differences in virome composition among samples may lead to different patterns of genome amplification in each sample. This may impact the results of our comparative analyses by introducing different biases in the samples that affect the observed patterns of microdiversity. Second, our metagenomes were prepared with the aim of extracting DNA solely from the double-stranded DNA (dsDNA) viruses in the samples. Hence, our results are representative only of viruses with that specific genomic composition, while other types, such as ssDNA viruses, albeit extremely abundant (44), were not assessed and their microdiversity was not evaluated. Third, most of our results are based on the *in silico* analysis of a single geographical location and with just one virome for each depth. Therefore, it is possible that some of the microdiversity variation explored was not captured in the single samples analyzed. Our analysis of the Tara Oceans and ALOHA data sets suggest that the observed trends of microdiversity are also present in other ecosystems. Nevertheless, studies with more samples and covering larger spatial gradients will be necessary to determine if the trends presented here are universal for marine viral communities.

Finally, we interpreted our results based on the assumption that each assembled scaffold is representative of a single given viral population. Nevertheless, it is possible that multiple populations of closely related viruses cooccur in these samples, which would mean that cross-population read mapping might have occurred. This means that the results of *pN/pS* ratios obtained are not representative of a specific population but instead represent whole-community patterns of mutation accumulation. Advances in single-virus genomics will likely circumvent these issues, allowing microdiversity to be evaluated at a per-individual level by differentiating among specific haplotypes (15).

### Concluding remarks.

Light, nutrient availability, and temperature are major factors structuring the taxonomic and functional compositions of marine viral communities (5, 9). These variables are major determinants of the energy available across the ecosystem, and they shift drastically throughout the depth gradient from which our samples were retrieved. However, in the Mediterranean Sea, temperature remains relatively constant below the seasonal thermocline (ca. 50 m) (10). Our data show that energy availability not only shapes the taxonomic compositions of viral communities but also influences how the genomes of these viruses accumulate mutations. The obtained results allowed us to postulate hypotheses about the selective pressures acting upon marine viruses from the community to the amino acid level. Furthermore, we demonstrated that the frequencies of nonsynonymous mutations differed among functional categories and depths. Finally, free-living viruses displayed patterns of mutation accumulation different from those observed under laboratory conditions, which has important implications for how the latter should be interpreted. Further research will be necessary to determine whether the patterns presented here are also present in different ecosystems (such as host-associated systems, freshwater, and soils) and to determine the driving forces behind them.

## MATERIALS AND METHODS

### Sampling and sample processing.

Four samples from different depths, 15, 45, 60, and 2,000 m, were collected on 15 October 2015 from aboard the research vessel *Garcia del Cid* (10). The sampling site was located approximately 60 nautical miles off the coast of Alicante, Spain, at 37.35361°N, 0.286194°W. Seawater samples were filtered for eukaryote and prokaryote fractions through 20-μm, 5-μm, and 0.22-μm-pore-size polycarbonate filters (Millipore). Two technical replicates (50 liters for each depth) were ultrafiltered on board through a Millipore Prep/Scale-TFF-6 filter, yielding 250 ml of viral concentrate stocks. Each stock was purified through 0.22-μm Sterivex filters (Millipore), stored at 4°C, and subsequently reduced to 1.5 ml using Ultra-15 centrifugal filter units (Amicon).

To minimize the carryover of free residual nucleic acids, stocks were treated with 2.5 U of DNase I at 37°C for 1 h, followed by inactivation with EDTA (0.5 mM). Total viral DNA was extracted with a PowerViral environmental RNA/DNA isolation kit (MO Bio). The quality and quantity of extracted DNA were determined using an ND-1000 spectrophotometer (NanoDrop, Wilmington, USA) and Qubit fluorometer (ThermoFisher). The absence of prokaryotic DNA was tested by PCR using 16S rRNA universal primers on aliquots from each sample. Multiple-displacement amplification (MDA) was performed using an Illustra GenomiPhi v2 DNA amplification kit (GE Healthcare, Life Sciences).

### Sequencing, assembly, and binning.

Metaviromes were sequenced using an Illumina Hiseq-4000 sequencer (150 bp, paired-end reads) by Macrogen (Republic of Korea). Reads from metaviromes were preprocessed using Trimmomatic (45) in order to remove low-quality bases (Phred quality score of 20 in 4-base sliding windows) and reads shorter than 30 bases. Each metagenome was individually assembled through SPAdes (46) using default parameters for the metagenomic mode. Sequences shorter than 5 kbp were discarded. Both raw reads and assembled scaffolds were deposited in the ENA under project number ERP113162. Cellular metagenomes have previously been published and deposited in a public repository as well (10). Taxonomic and functional annotations of proteins were performed by querying open reading frames (ORFs) against sequences in the NCBI nr database using DIAMOND (47).

Scaffolds from the cellular fraction of the 2,000-m sample were binned with MetaBat (48) to obtain metagenome-assembled genomes (MAGs) of bacteria and archaea. The quality of MAGs was assessed through CheckM (49). MAGs were manually curated to improve completeness and reduce potential contamination. ORFs were identified using the metagenomic mode of Prodigal (50).

### Identification of bona fide viral sequences.

A sequence-filtering step was performed to ensure that only bona fide viral sequences were included in downstream analyses. We considered viral sequences bona fide if they displayed a strong viral signal. This viral signal was assessed in three steps that relied on identifying sequence homology. First, ORFs from all scaffolds were queried against sequences in the Prokaryotic Virus Orthologous Groups (pVOGs) (51) database using HMMER (52) (E value ≤ 0.00001). Each orthologous group in the pVOGs database has a viral quotient that ranges from 0 to 1. Orthologous groups with a quotient of 1 are found exclusively in viral genomes and never in genomes of bacteria or archaea. The viral quotient decreases the more often the group is found in prokaryote genomes. Thus, for each scaffold assembled from the viromes, we calculated the percentage of ORFs mapped to the pVOGs database and their added viral quotient (AVQ; the sum of the individual viral quotients of the best hits of each protein). Sequences with 10% or more of their ORFs mapped to the pVOGs database and with an AVQ equal to or greater than 2 were classified as bona fide viruses, for a total of 2,937 scaffolds identified as viral sequences in this first round.

Second, the ORFs derived from the 2,937 scaffolds identified as viral in the first round were used as bait to identify more viral sequences. The ORFs from the remaining scaffolds were queried against those derived from first round using DIAMOND (47) (more sensitive mode, BLOSUM45 matrix, identity of ≥30%, bit score of ≥50, alignment length of ≥30 amino acids, and E value of ≤0.00001). Those scaffolds for which at least 20% of the derived ORFs matched (minimum of three matches) a single bona fide viral scaffold from the first round were also tagged as viral, for a total of 4,203 scaffolds. Third, we performed a step of manual curation, which recruited mostly long scaffolds with high AVQs that did not match the percentage criteria of the first-round steps due to their high number of ORFs, which identified 24 scaffolds as viral.

This three-step approach showed high concordance when the results were compared to the results of state-of-the-art tools used to identify viral sequences, namely, VirSorter (53) and VirFinder (54). Out of 7,164 sequences classified as bona fide viral scaffolds, only 166 (2.3%) were not identified as viral by either VirSorter (categories 1 to 6) or VirFinder (score ≥ 0.7 and *P* value ≤ 0.05). Thus, we conclude that our approach based on pVOGs, baiting, and manual inspection is highly accurate.

### Viral taxonomic affiliation and computational host prediction.

Putative hosts were assigned to viral scaffolds through homology matches, CRISPR spacers, and shared tRNAs as previously described (9). These were performed using two data sets: the NCBI RefSeq genomes of bacteria and archaea (June 2017 release) and the MAGs that were previously obtained from the binning of scaffolds from the same depth gradient from which the viromes were derived (10), plus the MAGs obtained from the binning of the 2,000-m samples (the only one that had not been previously binned). Putative hosts were manually assigned for sequences that displayed high similarity to RefSeq bacteriophage genomes as measured by the proportion of shared genes and synteny between genomes. Ambiguous host predictions, i.e., derived from viral sequences predicted to infect more than a single taxon, were removed from further analyses.

Taxonomic affiliations of viral sequences were determined by closest-relative affiliation. First, ORFs derived from the assembled scaffolds were queried against a subset of the NCBI nr database (containing viral sequences only) using DIAMOND (BLOSUM45 matrix, identity of ≥30%, bit score of ≥50, alignment length of ≥30 amino acids, and E value of ≤0.00001). Next, for each scaffold, we identified the taxon to which most ORFs were matched and defined it as the closest relative. This was performed for the taxonomic levels of family, genus, and species. In cases of ties (multiple taxa matching the same amount of ORFs), the closest relative was defined as the taxon with the highest value of average identity among matched ORFs.

### Abundance profiles and microdiversity analysis.

Sequencing reads from the cellular and viral metagenomes were mapped to assembled viral scaffolds using Bowtie2 in sensitive local mode (25). The number of reads mapped was used to estimate the relative abundances (i.e., percentages of mapped reads) of the viral sequences in both fractions. The relative abundance of each viral taxon was calculated by adding up the relative abundances of each scaffold according to their family-level taxonomic affiliation. Similarly, abundances of viral groups according to host were calculated by adding up the relative abundances of each scaffold according to their predicted host at the phylum level. The abundances of prokaryotes in the cellular metagenomes were determined by querying reads from these samples against the NCBI nr database using DIAMOND (47).

To estimate mutational frequencies of viral genomes, raw reads were mapped to assembled scaffolds using the sensitive local mode of Bowtie2. During read mapping, samples were sampled down to 50 million reads so that differences in metagenome coverage would not impact subsequent results. Next, the generated bam files were analyzed through DiversiTools (http://josephhughes.github.io/DiversiTools/) to obtain counts of synonymous and nonsynonymous mutations in each protein. Codon mutations were considered valid only if they were detected at least 4 times in at least 1% of the mapped reads and if the codon coverage was equal to or above 5×. Only the mutations that passed the aforementioned filters were considered to estimate the percentage of polymorphic sites and *pN/pS* ratios, which were calculated as described in reference 24.

### Microdiversity analysis in independent data sets.

We analyzed the microdiversity of samples from two independent data sets to contrast our results. Viromes from the Tara Oceans expeditions (station 64) were queried against a database of the Mediterranean Sea scaffolds. Read mapping and identification of polymorphic sites and calculation of *pN/pS* ratios were all performed exactly as described above. These samples were paired to our assembled scaffolds in the following way: SRA run ERR594392 (64_SRF, 5 m) with Mediterranean scaffolds from 15 m, ERR594385 (64_DMC, 65 m) with Mediterranean scaffolds from 45 m, and ERR599351 (64_MES, 1,000 m) with Mediterranean scaffolds from 2,000 m. Likewise, samples from the *Hawaii Ocean* time series (HOT-ALOHA) were paired with our assembled scaffolds in the following way: SRR5002342 (HOT224_1_0025m, 25 m) with Mediterranean scaffolds from 15 m, SRR5002371 (HOT224_1_0125m, 125 m, DCM) with Mediterranean scaffolds from 45 m, and SRR5002344 (HOT224_1_1000m, 1000 m) with Mediterranean scaffolds from 2,000 m.

### Availability of data.

The data sets supporting the conclusions of this article are available in the ENA repository (https://www.ebi.ac.uk/ena/data/view/PRJEB30684).
