# Supplementary figures and images for: Trends of Microdiversity Reveal Depth-Dependent Evolutionary Strategies of Viruses in the Mediterranean
Source: mSystems. 2019 Nov 5;4(6):e00554-19. doi: 10.1128/mSystems.00554-19 (PMC6832022; doi:10.1128/mSystems.00554-19)

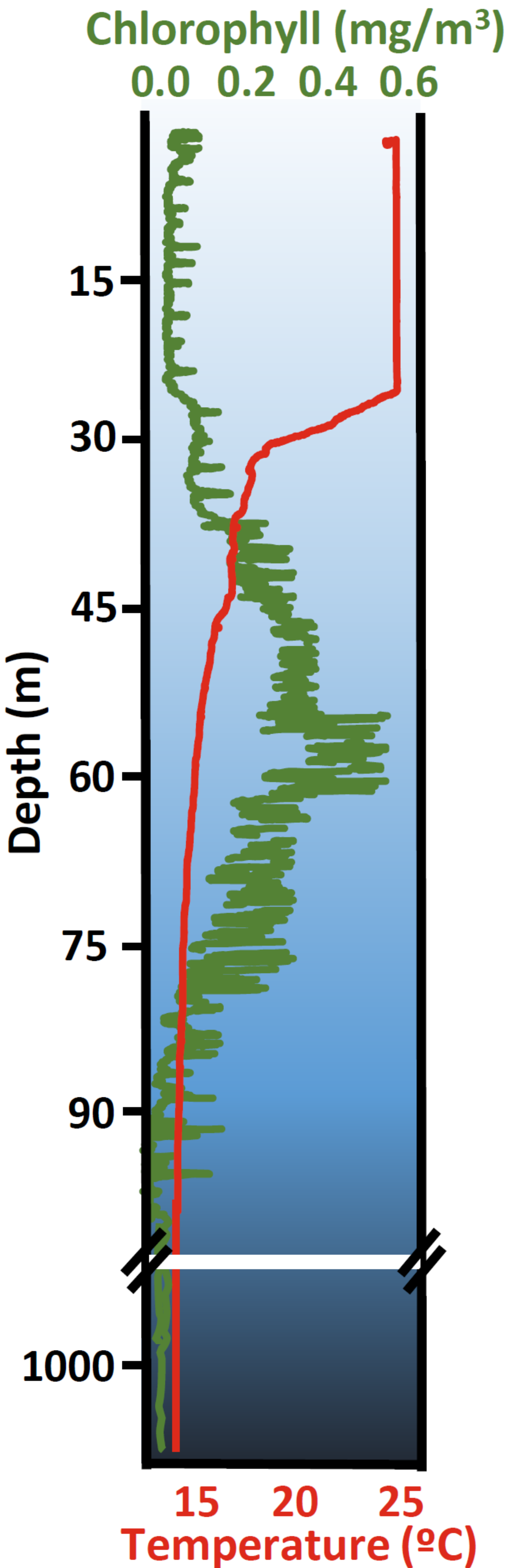

Supplement: FIG S1 [file mSystems.00554-19-sf001.pdf]

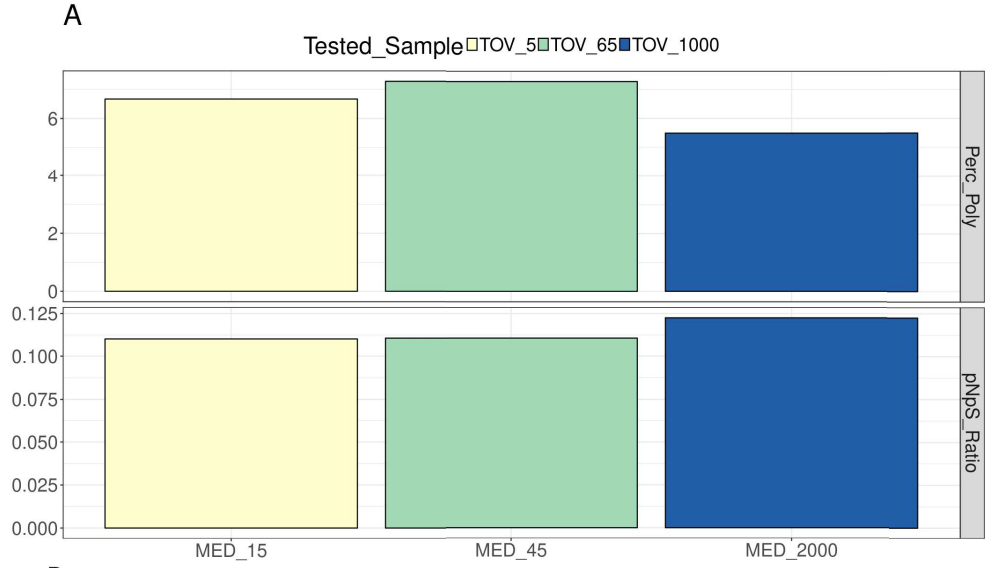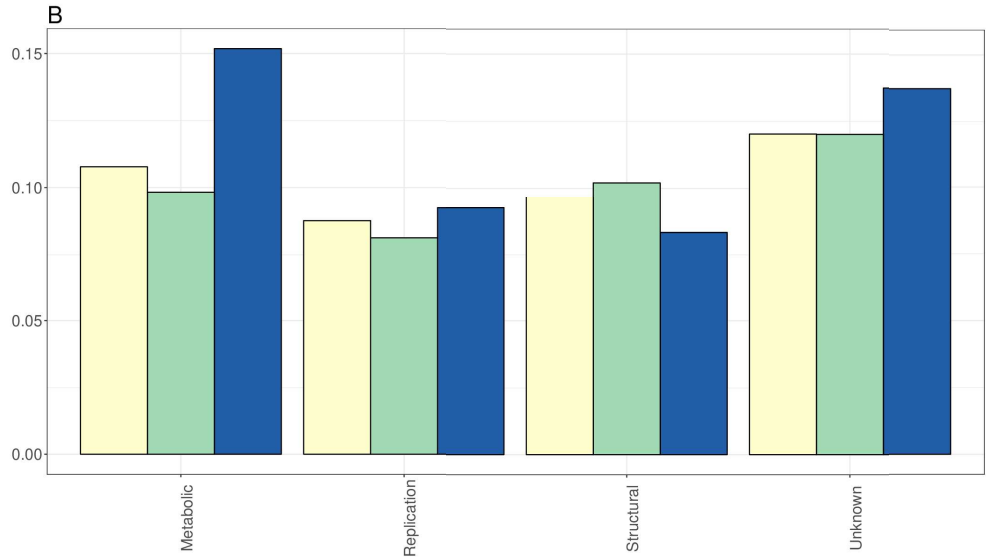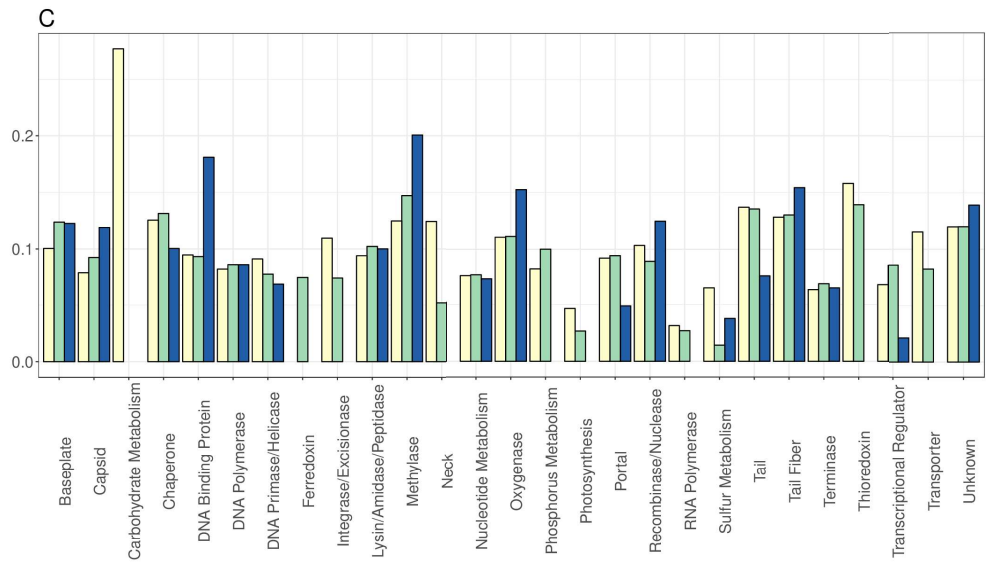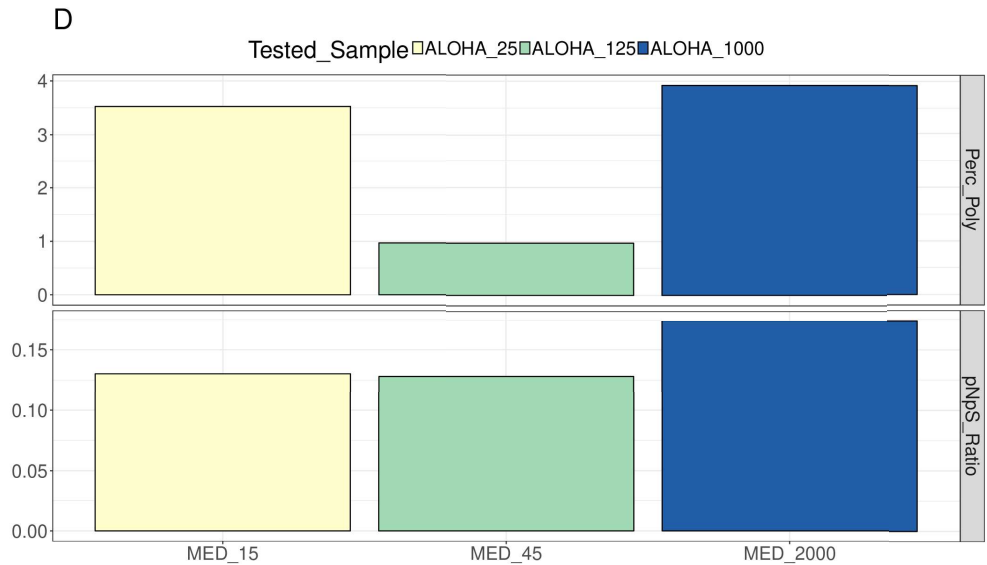

Supplement: FIG S2 [file mSystems.00554-19-sf002.pdf]
